# Structural insights into VAChT neurotransmitter recognition and inhibition

**DOI:** 10.1038/s41422-024-00986-5

**Published:** 2024-06-11

**Authors:** Yang Zhang, Fei Dai, Nanhao Chen, Dong Zhou, Chia-Hsueh Lee, Chen Song, Yixiao Zhang, Zhe Zhang

**Affiliations:** 1 State Key Laboratory of Membrane Biology, School of Life Sciences, Peking University, Beijing, China; 2 Interdisciplinary Research Center on Biology and Chemistry, Shanghai Institute of Organic Chemistry, Chinese Academy of Sciences, Shanghai, China; 3 State Key Laboratory of Chemical Biology, Shanghai Institute of Organic Chemistry, Chinese Academy of Sciences, Shanghai, China; 4 Center for Quantitative Biology, Academy for Advanced Interdisciplinary Studies, Peking University, Beijing, China; 5 Center for Life Sciences, Academy for Advanced Interdisciplinary Studies, Peking University, Beijing, China; 6 Department of Structural Biology, St. Jude Children’s Research Hospital, Memphis, TN USA

**Keywords:** Cryoelectron microscopy, Transport carrier

Dear Editor,

Acetylcholine (ACh) is an excitatory neurotransmitter with a wide variety of functions. In the central nervous system, ACh helps regulate memory, motivation, arousal, and attention. ACh controls muscle contraction, blood pressure, intestinal peristalsis, and glandular secretion in the peripheral nervous system. Decreased ACh signaling is strongly related to multiple pathological conditions, such as Alzheimer’s disease (AD), Lambert–Eaton myasthenic syndrome, and myasthenia gravis.^1^ ACh is synthesized in the cytosol, and its active transport into synaptic vesicles is mediated by vesicular acetylcholine transporter (VAChT or SLC18A3).^2^ Remarkably, VAChT plays a vital role in ACh neurotransmission. The vesicular accumulation of ACh is necessary for an efficient secretion of neurotransmitters in response to upstream Ca^2+^ signals. Knockout or knockdown of *VAChT* in mice severely impairs neuromuscular and cognitive functions.^3,4^ Furthermore, genetic variants in VAChT can cause a congenital myasthenic syndrome in humans.^5^ Like other vesicular neurotransmitter transporters, VAChT is a proton antiporter that pumps ACh into synaptic vesicles through the coupled efflux of protons.^6^ Currently, vesamicol is the best-characterized inhibitor of VAChT with nanomolar affinity.^7^ Vesamicol is used for functional studies on VAChT and as a specific marker of the cholinergic system. Given that AD is typically associated with the degeneration of cholinergic neurons, radiolabeled analogs of vesamicol have the potential for application in the early diagnosis of AD.^8^ To investigate the inhibitory mechanism of vesamicol and guide subsequent efforts to develop drugs, we determined the cryo-electron microscopy (cryo-EM) structure of human VAChT in complex with vesamicol or its native substrate ACh. In both structures, VAChT adopts a lumen-facing conformation. Although vesamicol and ACh share a conserved binding mode, they each occupy a unique pocket. Our mutagenesis studies further pinpointed the critical residues of VAChT essential for vesamicol recognition. Overall, our work provides a structural framework for clarifying how VAChT recognizes its substrates and inhibitors.

To assist in the structure determination, we attached a maltose binding protein (MBP) tag to the N-terminus of human VAChT and an MBP-specific binder, a designed ankyrin repeat protein (DARPin), to its C-terminus; this strategy was similar to that reported in our structural study on VMAT2 (Fig. 1a; Supplementary information, Fig. S1a).^9^ These modifications had little effect on the binding of vesamicol (Supplementary information, Fig. S1b, VAChT^EM^ construct). Subsequently, we determined the cryo-EM structure of the VAChT^EM^/vesamicol complex at a 3.5 Å resolution by local refinement, which was focused on the transporter portion (Fig. 1b; Supplementary information, Figs. S1c, S2, and Table S1). In this structure, VAChT adopts a major facilitator superfamily fold in a lumen-facing conformation (Fig. 1b; Supplementary information, Fig. S1d). The two lobes of transmembrane (TM) domains (TM1–6 and TM7–12) open to the luminal side, similar to the reported lumen-facing serotonin (5-HT)- and ketanserin-bound VMAT2 structures (Supplementary information, Fig. S1e).^10^ On the cytosolic side, the intracellular gate between TM4–5 and TM10–11 is tightly sealed mainly by two layers of hydrophobic interactions. Specifically, Ile197^TM4^, Leu214^TM5^, Leu402^TM10^ and Tyr421^TM11^ surround the inner layer, whereas Ala201^TM4^, Arg210^TM5^, Ala406^TM10^, and Tyr417^TM11^ are located in the outer layer. In addition, Arg210 forms hydrogen bonds with Tyr417 and the main-chain carboxyl group of Ala406, further stabilizing this gate (Fig. 1c). Overall, the architecture of the intracellular gate resembles that of VMAT2,^9^ with all key residues conserved across species and within the three SLC18A family transporters (Supplementary information, Fig. S3). On the luminal side, the extracellular gate is largely open. However, importantly, the side chains of Met51 and Glu309 create a barrier in the passageway to the vesicle lumen and partially block the pathway (Fig. 1d). This finding suggested that these two residues are involved in finely tuning the access of substrates to the central binding site of VAChT.

**Fig. 1:**
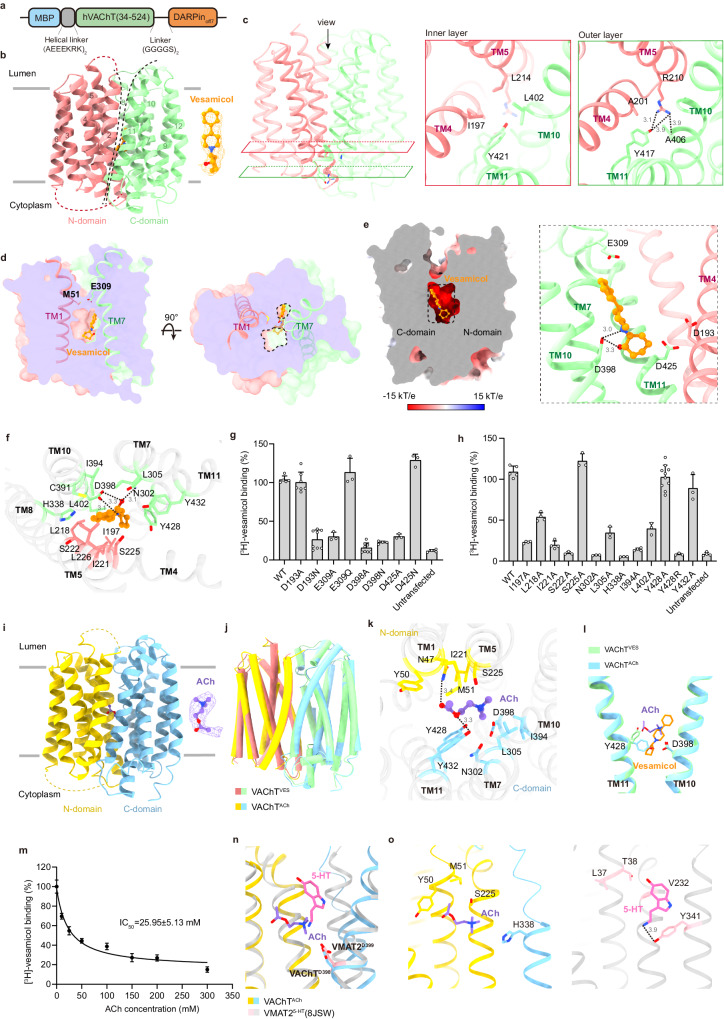
Molecular mechanisms for vesamicol and ACh recognition by VAChT. **a** Schematic diagram of MBP-VAChT-DARPin. **b** Structure of vesamicol-bound VAChT in the lumen-facing conformation. **c** The cytosolic gate of VAChT. Two layers of gate residues from TMs 4, 5, 10, and 11 are shown as sticks on the side in magnified views. **d** Slice view of the vesamicol-bound VAChT structure. Vesamicol is shown in dark orange. TM1 and TM7 are shown as cartoon representations. **e** Left, the electrostatic surface potential of the vesamicol binding site calculated with the program APBS (http://www.poissonboltzmann.org); right, locations of the four acidic residues around vesamicol. **f** Details of the interaction between VAChT and vesamicol. The dashed lines represent hydrogen bonds, salt bridges, or electrostatic interactions (< 4.0 Å). The corresponding distances are indicated in angstroms. The residues involved in van der Waals and hydrophobic interactions (< 4.5 Å) are shown with side chains. **g**, **h** Binding of [^3^H]-vesamicol to different VAChT variants. One hundred percent binding was defined as the average [^3^H]-vesamicol signal bound to wild-type (WT) VAChT. The data are shown as the means ± SD; *n* = 3–10 biological replicates. **i** Structure of the ACh-bound VAChT. **j** Comparison of the structures of vesamicol- and ACh-bound VAChT. The root-mean-square deviation between these two structures is 0.9 Å. **k** Details of the interaction between VAChT and ACh. **l** Comparison of the ACh- and vesamicol (VES)-binding sites in VAChT. For clarity, only TM10 and TM11 are shown in pale green (vesamicol-bound) or light sky blue (ACh-bound). Asp398 and Tyr428 are shown as sticks. **m** Competitive binding analyses of ACh and [^3^H]-vesamicol with VAChT. One hundred percent binding was defined as the average [^3^H]-vesamicol signal bound to VAChT in the absence of ACh. The data are shown as the means ± SD; *n* = 3 biological replicates. **n**, **o** Comparison of the binding modes of ACh in VAChT and 5-HT in VMAT2 (**n**). The nonconserved residues within the substrate binding sites are shown in **o**.

The additional EM density in the central cavity of VAChT fits well with the structure of D-(+)-vesamicol (Fig. 1b), whose binding pose was further validated by molecular dynamics (MD) simulations (Supplementary information, Fig. S4a, b). Vesamicol binds to a negatively charged pocket of VAChT, which can accommodate the positively charged alkylamine group in vesamicol (Fig. 1e). Notably, Asp398 is directly involved in the electrostatic interaction with the piperidinyl nitrogen of vesamicol. Additionally, the following acidic residues are strategically positioned: Glu309 is above the phenyl group, while Asp193 and Asp425 are close to the cyclohexanol moiety (Fig. 1e). Moreover, the hydroxyl group of the cyclohexanol ring is engaged by Asn302 and Asp398 via hydrogen bonds, further stabilizing the binding of vesamicol (Fig. 1f; Supplementary information, Fig. S1f, g). In addition to electrostatic and hydrophilic interactions, vesamicol extensively contacts multiple residues of VAChT through hydrophobic or van der Waals interactions (Fig. 1f; Supplementary information, Fig. S1f, g). For example, Ser222, Ser225, Leu226, Leu305, His338, Cys391, and Ile394 pack against the phenyl ring. On the other hand, Ile197, Leu218, Ile221, Leu402, Tyr428, and Tyr432 complement the hydrophobic skeleton of the piperidinyl and cyclohexanol moieties.

To verify the vesamicol binding site, we individually mutated the surrounding residues observed in our structure and examined the [^3^H]-vesamicol binding ability of these mutants in HEK293F cells. Our results showed that the substitution of Asp398 with alanine or asparagine nearly completely abolished the binding of vesamicol, consistent with its key role in electrostatic interactions and previous results^11,12^ (Fig. 1g; Supplementary information, Fig. S5a). Interestingly, unlike the E309A and D425A mutations, which disrupt vesamicol binding, the E309Q and D425N mutations of VAChT had no effect (Fig. 1g; Supplementary information, Fig. S5a). This result suggests that the polar nature of these two sites, rather than the charge, contributes to the formation of the binding site. In contrast to these two residues, replacing Asp193 with asparagine, but not alanine had a greater impact on vesamicol binding. One possible explanation is that the protonation of Asp193 might destabilize the current structure, thereby weakening the binding of vesamicol (Fig. 1g; Supplementary information, Fig. S5a). This finding aligns with a previous study in which Asp193 was potentially involved in the proton-coupled conformational transition during the transport cycle of VAChT.^13^ Furthermore, mutating most of the other residues around the binding site to alanine severely reduced the affinity of vesamicol (Fig. 1h; Supplementary information, Fig. S5b), underscoring the importance of the overall environment for the recognition of vesamicol. It is worth noting that there are extra spaces around the vesamicol binding pocket, especially near the cyclohexanol group (Supplementary information, Fig. S1h). This suggests that additional modifications of vesamicol might be feasible to further increase its potency. Consistent with this notion, several vesamicol analogs with proper alterations to the cyclohexanol ring indeed exhibited greater affinities for VAChT, such as benzovesamicol and trozamicol derivatives.^8,14^ It remains elusive whether vesamicol exerts its inhibitory effect on VAChT by competing with the substrate or in an allosteric manner.^7,15^ To investigate this, we further determined the structure of VAChT^EM^ in complex with ACh at 3.7 Å resolution (Fig. 1i; Supplementary information, Figs. S6, S7a, and Table S1). In this structure, VAChT adopts a similar conformation to that observed when VAChT binds vesamicol (Fig. 1j). The binding site and pose of ACh only roughly agreed with the MD simulation results (Supplementary information, Fig. S4c), implying that the ACh association is relatively dynamic in the current conformation. This is consistent with the fact that ACh is about to be released in the lumen-facing state of VAChT. Although both ACh and vesamicol occupy the central pocket of VAChT, their orientations differ: ACh lies parallel to the membrane, with its acetyl group facing the N-lobe of VAChT and its choline group pointing to the C-lobe (Fig. 1k, l). Most notably, the ammonium cation of ACh is located in a similar position to the piperidinyl amine of vesamicol, which could also potentially interact weakly with Asp398 (4.8 Å away) (Fig. 1l). This finding highlights a conserved recognition pattern for ACh and vesamicol by VAChT. However, compared to that of vesamicol, the main body of ACh extends into a distinct binding site (Fig. 1k, l). In summary, ACh not only engages some residues identical to those of vesamicol, such as Ile221, Ser225, Asn302, Leu305, Ile394, Asp398, Tyr428, and Tyr432, but also contacts three new residues via its acetyl group, namely Asn47, Tyr50, and Met51 (Fig. 1k; Supplementary information, Fig. S7b, c). Although most key residues maintain similar side chain rotamers in both the ACh- and vesamicol-bound structures, Tyr428 notably flips in different directions to better accommodate each specific molecule (Fig. 1f, k, l). The partially overlapping binding sites of these two molecules in our structures suggest that vesamicol can competitively inhibit the substrate binding and transport activity of VAChT (Fig. 1l). This result is supported by the weak ability of ACh to inhibit the binding of vesamicol to VAChT, with a high IC_50_ value of 26 mM (Fig. 1m). In addition to its competitive nature, vesamicol may bind and stabilize VAChT in its lumen-facing conformation, thereby arresting the transport cycle; this process is akin to how tetrabenazine inhibits VMAT2.^9^ In this context, vesamicol functions partially as an allosteric inhibitor.

Unlike VAChT, the other two members of the SLC18A subfamily, VMAT1 (SLC18A1) and VMAT2 (SLC18A2), transport monoamines but not ACh. Strikingly, in the 5-HT-bound VMAT2 structure, the amine group of 5-HT is located in a similar position to those of vesamicol and ACh (Fig. 1l, n). Therefore, all three SLC18A members recognize the amine group of their substrates in a similar manner using a conserved acidic residue (Asp398 in VAChT, Asp407 in VMAT1, and Asp399 in VMAT2) (Fig. 1l, n; Supplementary information, Fig. S3). How, then, do VAChT and VMATs distinguish their substrates? From the structural comparison, we observed that the main bodies of ACh and 5-HT occupy different pockets. Within their respective binding sites, four residues are not conserved between VAChT and VMATs (Fig. 1o; Supplementary information, Fig. S3). Among these residues, Tyr50, Met51, and Ser225 of VAChT participate in the binding of ACh, while their cognate residues in VMATs likely do not perform the same function (Fig. 1o). Similarly, Val232 and Tyr341 of VMAT2 (corresponding to Leu240 and Tyr349 of VMAT1, respectively) play pivotal roles in 5-HT and dopamine transport,^9,10^ whereas these two residues are replaced by serine (Ser225) and histidine (His338), respectively, in VAChT (Fig. 1o). Therefore, it is plausible that these differences may contribute to the substrate specificity observed among different SLC18A family proteins.

Taken together, the results of this study reveal the distinct binding patterns of vesamicol and ACh to VAChT, providing new insights into the mechanism by which vesamicol exerts activity. Our structures here could well explain some previous mutagenesis data. For example, it is reported that mutation D398H can cause congenital myasthenic syndrome.^5^ This is very likely because it disrupts the substrate recognition of VAChT (Fig. 1k). Likewise, according to our structures, Cys391 is only involved in vesamicol binding but not in ACh binding (Fig. 1f, k). Therefore, mutating it to tyrosine (C391Y) would only abolish vesamicol sensitivity but not affect the ACh transport activity of VAChT.^15^ Furthermore, our structural information offers important guidance for developing inhibitors and drugs targeting VAChT. For instance, appropriate vesamicol analogs that can adequately occupy its binding pocket (Supplementary information, Fig. S1h) are likely to exhibit increased affinity for VAChT.

### Supplementary information

Supplementary Information

## Data Availability

Cryo-EM density maps of vesamicol- and ACh-bound VAChT were deposited in the Electron Microscopy Data Bank under the accession codes EMD-60254 and EMD-60255, respectively. Their atomic coordinates have been deposited in the Protein Data Bank under accession codes 8ZMR and 8ZMS, respectively.
