# Supplementary material for: Structural insights into VAChT neurotransmitter recognition and inhibition
Source: Cell Res. 2024 Jun 11;34(9):665–8. doi: 10.1038/s41422-024-00986-5 (PMC11369146; doi:10.1038/s41422-024-00986-5)
Supplement: Supplementary file 1 — Supplementary Information [file 41422_2024_986_MOESM1_ESM.pdf]

Supplementary Information

Structural insights into VAChT neurotransmitter recognition and inhibition

Authors:

Yang Zhang[1,7], Fei Dai[2,3,7], Nanhao Chen[4,7], Dong Zhou[5], Chia-Hsueh Lee[6*], Chen Song[4,5*], Yixiao Zhang[2,3*] and Zhe Zhang[1,5*]

Affiliations:

[1]State Key Laboratory of Membrane Biology, School of Life Sciences, Peking University, Beijing 100871, China

[2]Interdisciplinary Research Center on Biology and Chemistry, Shanghai Institute of Organic Chemistry, Chinese Academy of Sciences, Shanghai 200032, China.

[3]State Key Laboratory of Chemical Biology, Shanghai Institute of Organic Chemistry, Chinese Academy of Sciences, Shanghai 200032, China.

[4]Center for Quantitative Biology, Academy for Advanced Interdisciplinary Studies, Peking University, Beijing 100871, China

[5]Center for Life Sciences, Academy for Advanced Interdisciplinary Studies, Peking University, Beijing 100871, China

[6]Department of Structural Biology, St. Jude Children's Research Hospital, Memphis, TN 38105, United States

[7]These authors contributed equally: Yang Zhang, Fei Dai, Nanhao Chen.

*Correspondence should be addressed to chiahsueh.lee@stjude.org, c.song@pku.edu.cn, yzhang@mail.sioc.ac.cn, or zzhang01@pku.edu.cn.

Methods

Cell culture

HEK293S GnTI$^-$ cells were maintained in Freestyle 293 Expression Medium (Gibco). Medium was supplemented with 1% fetal bovine serum (FBS). HEK293F cells were cultured in serum- and antibiotic-free SMM 293-TII medium (Sino Biological Inc.). Sf9 cells were maintained in Sf-900 II SFM medium (Gibco). Mammalian cells were cultured at 37 °C with 5% CO_2. Sf9 cells were cultured at 28 °C.

Expression and purification of VAChT^{EM}

The DNA sequence of human VAChT (residues 34-524) was cloned into a pEG BacMam vector (TransGen). The coding sequence of MBP and a helical linker $(AEEEKRK)_2$ were inserted into the N terminus of VAChT, and a $(GGGGS)_2$ linker together with the coding sequence of DARPin$_{off7}$ was attached to the C terminus. This construct was referred to as the VAChT^{EM} hereafter.

VAChT^{EM} was expressed in HEK293S GnTI$^-$ cells using BacMam system[1]. Baculoviruses were produced by transfecting Sf9 cells. After three rounds of amplification, viruses were used for cell transduction. HEK293S cells at 3×10^6 cells/ml were infected by adding 10% 3rd passage baculoviruses and cultured at 37 °C for 8-12 h. Then, 10 mM sodium butyrate was added to induce protein expression. Meanwhile, cells were transferred to 30 °C and cultured for another 48 h. Cells were collected and stored at −80 °C.

For protein purification, frozen cell pellets were thawed at room temperature and then resuspended in lysis buffer (50 mM HEPES pH 7.25, 300 mM NaCl, and 15% glycerol) supplemented with 2 μg/ml DNase I and protease inhibitor cocktail (APExBIO), then cells were

lysed with 2% n-dodecyl β-D-maltoside (DDM) and 0.2% Cholesteryl hemisuccinate (CHS) at 4 °C for 3 h. After centrifugation at 18,000 rpm for 40 min, the soluble fraction was incubated with anti-GFP nanobody affinity resin at 4 °C for 2 h. The resin was then washed with 40 column volumes of Buffer A (25 mM HEPES pH 7.25, 150 mM NaCl, and 0.02% DDM-0.002% CHS). GST-tagged PreScission Protease was applied to remove the GFP-tag and release $VAChT^{EM}$ from the resin. The protease was removed by binding GSH affinity beads (Smart-Life sciences). The protein was further processed by size-exclusion chromatography (SEC) using a Superose 6 Increase 10/300 GL column (GE Healthcare). The peak fractions were concentrated with a 100 kDa cut-off centrifugal filter (Millipore) to around 10 mg/ml. The protein sample was stored at −80 °C for later use.

Cryo-EM sample preparation and data collection

For the $VAChT^{EM}$/vesamicol complex, the purified protein was incubated with 100 μM vesamicol (Sigma) at 4 °C for 30 min before preparing the cryo-EM sample. For the $VAChT^{EM}$/ACh complex, the protein was incubated with 100 mM acetylcholine (Sigma). 3 μl protein sample was applied to glow-discharged holey carbon grids (Quantifoil R1.2/1.3 Au300). The grids were flash frozen in liquid ethane cooled by liquid nitrogen using a Vitrobot Mark IV (FEI) at 10 °C and 100% humidity. The blotting parameters were set at the blot time of 3 s and the wait time of 10 s.

The grids were initially screened using a 200 kV Talos Arctica microscope (FEI) equipped with a Gatan K2 Summit detector. Raw movie stacks were recorded using a 300 kV Titan Krios microscope (FEI) with a K3 camera (Gatan) at a physical pixel size of 0.83 Å per pixel and a nominal defocus range of 1.0–2.0 μm. Each movie contained 40 frames. The total exposure dose

was about 60 $e^{-}/Å^2$ and the exposure time was 3.2 s. The data collection parameters are summarized in Supplementary information, Table S1.

Cryo-EM image processing

The image stacks were gain-normalized and corrected for beam-induced motion using Patch Motion Correction in cryoSPARC[2]. The CTF parameters were estimated using Patch CTF Estimation. Micrographs not suitable for further analysis were removed by manual inspection.

For the vesamicol-bound dataset, 500 micrographs were used for initial particle picking and generation of the 2D templates. Subsequently, particles were picked out using these 2D templates as reference by template picking and for training in the Topaz particle-picking pipeline[3]. After two rounds of 2D classification, selected particles were used for ab initio reconstructions and heterogeneous refinements. Then, the particles from the good classes were combined, and duplicated particles were removed. Iterative 3D classifications were then performed with subsequent ab initio reconstructions and heterogeneous refinements to remove suboptimal particles. Selected particles were refined with nonuniform refinement[4]. These particles were used as seeds for the subsequent seed-facilitated guided heterogeneous refinement performed on the particles picked with lower threshold[5]. Then, the low passed map were used for heterogeneous refinement, and the selected particles were refined with nonuniform refinement. A following local refinement of only the transporter portion produced a 3.5 Å-resolution map using the gold-standard FSC = 0.143. A detailed flowchart of the data process is presented in Supplementary information, Fig. S2.

For the acetylcholine-bound dataset, all the micrographs were used for blob picking and generation of the 2D templates. After 4 rounds of 2D classification, the selected particles were

used for ab initio reconstructions and heterogeneous refinements. Subsequently, the particles from the good classes were used as template for template picking and training in the Topaz particle-picking pipeline. Each set of particles were classified by several rounds of 2D classification and heterogeneous refinements. Subsequently, the particles were combined and duplicated particles were removed. After removing the suboptimal particles by ab initio reconstructions and heterogeneous refinements, the particles from the best class were input as seed for seed-facilitated guided heterogeneous refinement. Then, the low passed map were used for heterogeneous refinement, and the selected particles were refined with nonuniform refinement. A following local refinement of only the transporter portion produced a 3.7 Å-resolution map using the gold-standard FSC = 0.143. A detailed flowchart of the data process is presented in Supplementary information, Fig. S6.

Model building and refinement

The AlphaFold-predicted model of VAChT[6] was roughly fitted into the vesamicol-bound VAChT map using ChimeraX[7]. Then, real-space refinement was carried out using PHENIX[8], and manual adjustment was done in Coot[9]. Alternative automatic and manual refinements were performed before obtaining the final structural model. This VAChT structure was used for the model building and refinement of the ACh-bound structure. Local resolutions of the cryo-EM maps were estimated using cryoSPARC. Geometries of the structure models were validated by MolProbity[10]. All the structure related figures were generated using ChimeraX.

Molecular Dynamics (MD) Simulations

The molecular dynamics simulations were started from the Cryo-EM models resolved in this work. The residue Phe266 and the residue Leu276 were appended and prepended respectively to the missing loop between TM6 and TM7, avoiding prolines (Pro265 and Pro277) as the termini. All the other missing residues were ignored since they were located outside the membrane. PROPKA3[11] was used to estimate the pK_a values of the residues. As displayed in Fig. S4a, the acidic residues were classified into three groups based on their locations. The lumen-facing acidic residues (shown in blue bars) and the cyto-facing acidic residues (shown in yellow bars) were all deprotonated because the predicted pK_a values were lower than the corresponding environmental pH values. The protonation states of the buried residues were determined based on the pK_a values and the surrounding residues (hydrogen-bond networks). Herein, Asp46 and Glu309 were designated as protonated while the others were deprotonated.

The MD simulation systems were set up by the CHARMM-GUI server[12, 13]. Here, the N- and C-terminus of all chains were capped by ACE (acetyl) and NME (N-methylamide), and the protein and lipid molecules were described by the CHARMM36m force field[14]. The ligands in the systems were described by the CHARMM all-atom forcefield[15]. The transmembrane region of the protein was calculated by the PPM web server[16] and the DMPC lipids were added to form a bilayer membrane. Two layers of TIP3P water molecules[17] were added above and below the bilayer membrane, with a thickness of 22.5 Å, respectively. Chloride ions were added to the system to neutralize the simulation system.

The MD simulations started from the structure that was energy minimized with position restraints on the protein, ligand, and lipids (10, 10, and 2.5 kcal/mol/Å^2 respectively). Next, two-step NVT simulations were conducted to heat the system to 300 K in 250 ps. The position restraints on protein and ligand were set to 10 kcal/mol/Å^2 in the first step and later reduced to 5, while the

lipids were restrained with a force constant of 2.5 kcal/mol/$Å^2$. Similarly, multi-step NPT simulations were carried out to equilibrate the system while loosening the restraints of the protein and lipids gradually. Finally, a 200-ns MD simulation under the NPT ensemble was performed. The time step of the simulations was set to 1 fs for the heating and equilibrium processes, and set to 2 fs for the final production MD simulations. During the simulations, the Langevin thermostat with a 1/ps friction coefficient was applied to control the temperature of the system, and the Monte Carlo barostat[18] was used to control the semi-isotropic pressure. The nonbonding interactions were turned off with a switching function from 10 to 12 Å, while the particle-mesh Ewald (PME) method was chosen to describe the long-range summation of the electrostatic interactions. In addition, the SHAKE algorithm[19] was used to constrain bonds involving hydrogen. After the MD simulations, MM/GBSA calculations[20] were performed to calculate the protein-ligand binding free energies for the last 20 ns trajectories. All the simulations were done with AMBER20[21].

[^{3}H]-vesamicol binding assay

Radioligand binding assays were done using crude cell membranes containing VAChT. HEK293F cells transiently transfected with VAChT plasmids were cultured at 37 °C for 16–18 h followed by the addition of 10 mM sodium butyrate and cultured at 30 °C for another 24 h. Cells were washed with cold PBS and resuspended in 500 µl cold SH buffer (320 mM sucrose, 10 mM HEPES pH 7.4, and protease inhibitor cocktail) to a density of nearly 2×10^7 cells/ml. Cells were lysed by sonication. The cell lysate was centrifuged at 4000 g for 5 min to remove unlysed cells and debris. The supernatant was considered as crude membrane fraction. The prepared membranes were frozen in liquid nitrogen and stored in −80 °C.

For $[^3H]$-vesamicol binding assay, the membrane suspension (50 µl) was mixed with binding buffer (150 µl) containing 110 mM potassium tartrate, 20 mM HEPES (pH 7.4), 1 mM ascorbic acid, 25 nM vesamicol and 3 nM $[^3H]$-vesamicol (30-60 Ci/mmol, American Radiolabeled Chemicals, Inc.). After incubation for 60 min at 25 °C, the binding reaction was terminated by vacuum filtration through GF/C glass fibers filters (Whatman) presoaked in 0.3% polyethyleneimine, followed by a 2-ml wash with ice-cold binding buffer. Radioactivity bound to the filters was measured by scintillation counting in 3 ml scintillation solution (PerkinElmer, USA).

The competition assay of $[^3H]$-vesamicol binding by ACh was done similarly except for the following: the membrane suspension (50 µl) was incubated with indicated concentrations of acetylcholine for 20 min at 25 °C. This was followed by the addition of 150 µl binding buffer containing 25 nM vesamicol and 3 nM $[^3H]$-vesamicol and incubated for 20 min at 25 °C. The IC_{50} values were determined by fitting three-parameter dose-response curves using GraphPad Prism 9 (http://www.graphpad.com).

For comparing the binding among VAChT variants, the GFP fluorescence intensity of each construct was measured using fluorescence-detection size exclusion chromatography (FSEC), and the intensity was divided by that of the wild type to determine the differences in expression levels (Supplementary information, Fig. S5). These ratios were then applied to normalize the binding signal for each mutant.

Supplementary References

1. Goehring, A. *et al.* Screening and large-scale expression of membrane proteins in mammalian cells for structural studies. *Nature protocols* **9**, 2574-2585 (2014).
2. Punjani, A., Rubinstein, J.L., Fleet, D.J. & Brubaker, M.A. cryoSPARC: algorithms for rapid unsupervised cryo-EM structure determination. *Nature methods* **14**, 290-296 (2017).
3. Bepler, T. *et al.* Positive-unlabeled convolutional neural networks for particle picking in cryo-electron micrographs. *Nature methods* **16**, 1153-1160 (2019).
4. Punjani, A., Zhang, H. & Fleet, D.J. Non-uniform refinement: adaptive regularization improves single-particle cryo-EM reconstruction. *Nature methods* **17**, 1214-1221 (2020).
5. Wang, N. *et al.* Structural basis of human monocarboxylate transporter 1 inhibition by anti-cancer drug candidates. *Cell* **184**, 370-383. e313 (2021).
6. Jumper, J. *et al.* Highly accurate protein structure prediction with AlphaFold. *Nature* **596**, 583-589 (2021).
7. Goddard, T.D. *et al.* UCSF ChimeraX: Meeting modern challenges in visualization and analysis. *Protein science* **27**, 14-25 (2018).
8. Afonine, P.V. *et al.* Real-space refinement in PHENIX for cryo-EM and crystallography. *Acta Crystallographica Section D: Structural Biology* **74**, 531-544 (2018).
9. Emsley, P., Lohkamp, B., Scott, W.G. & Cowtan, K. Features and development of Coot. *Acta Crystallographica Section D: Biological Crystallography* **66**, 486-501 (2010).
10. Davis, I.W. *et al.* MolProbity: all-atom contacts and structure validation for proteins and nucleic acids. *Nucleic Acids Res* **35**, W375-W383 (2007).

11. Olsson, M.H., Søndergaard, C.R., Rostkowski, M. & Jensen, J.H. PROPKA3: consistent treatment of internal and surface residues in empirical p K a predictions. *Journal of chemical theory and computation* **7**, 525-537 (2011).

12. Jo, S., Klauda, J.B. & Im, W. CHARMM-GUI Membrane Builder for Mixed Bilayers and Its Application to Yeast Membranes. *Biophys J* **96**, 41a-41a (2009).

13. Park, S., Choi, Y.K., Kim, S., Lee, J. & Im, W. CHARMM-GUI Membrane Builder for Lipid Nanoparticles with Ionizable Cationic Lipids and PEGylated Lipids. *J Chem Inf Model* **61**, 5192-5202 (2021).

14. Huang, J. *et al.* CHARMM36m: an improved force field for folded and intrinsically disordered proteins. *Nature Methods* **14**, 71-73 (2017).

15. Venable, R.M. *et al.* CHARMM All-Atom Additive Force Field for Sphingomyelin: Elucidation of Hydrogen Bonding and of Positive Curvature. *Biophys J* **107**, 134-145 (2014).

16. Lomize, M.A., Pogozheva, I.D., Joo, H., Mosberg, H.I. & Lomize, A.L. OPM database and PPM web server: resources for positioning of proteins in membranes. *Nucleic Acids Res* **40**, D370-D376 (2012).

17. Jorgensen, W.L., Chandrasekhar, J., Madura, J.D., Impey, R.W. & Klein, M.L. Comparison of Simple Potential Functions for Simulating Liquid Water. *J Chem Phys* **79**, 926-935 (1983).

18. Bernetti, M. & Bussi, G. Pressure control using stochastic cell rescaling. *J Chem Phys* **153**, 114107 (2020).

19. Macuglia, D. SHAKE and the exact constraint satisfaction of the dynamics of semi-rigid molecules in Cartesian coordinates, 1973-1977. *Arch Hist Exact Sci* **77**, 345-371 (2023).

20. Wang, E.C. *et al.* End-Point Binding Free Energy Calculation with MM/PBSA and MM/GBSA: Strategies and Applications in Drug Design. *Chem Rev* **119**, 9478-9508 (2019).

21. D.A. Case. *et al.* AMBER 2020. University of California, San Francisco (2020).

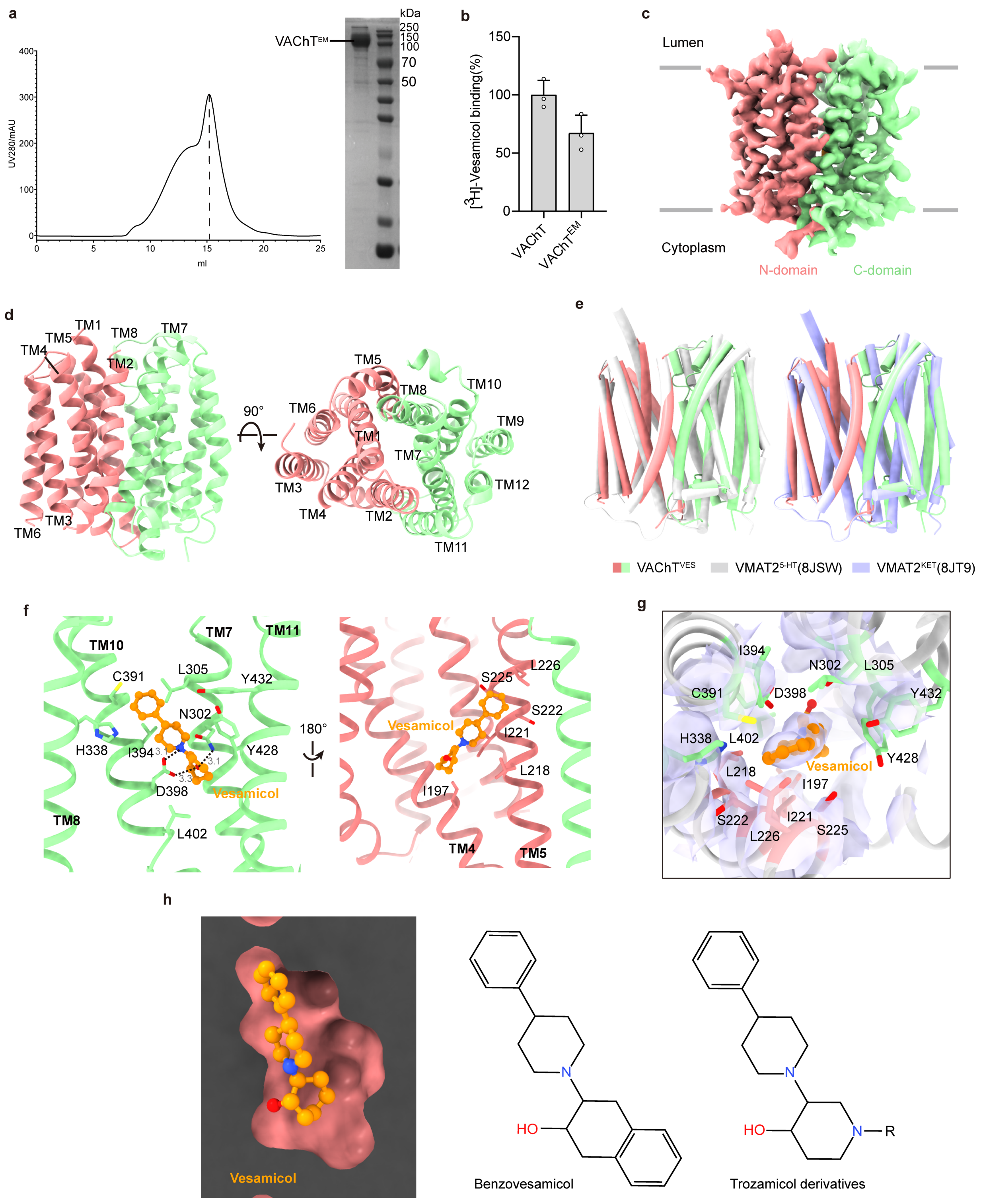

Fig. S1 Molecular mechanism of vesamicol recognition.

a Profile of the size exclusion chromatography (SEC) and SDS–PAGE results. **b** Binding of [^{3}H]-vesamicol to wild-type (WT) VAChT or VAChT^{EM}. One hundred percent binding was defined as

the average [^{3}H]-vesamicol signal bound to WT VAChT. The data are shown as the means ± s.d.; n = 3 biological replicates. **c** Cryo-EM map of the VAChT/vesamicol complex. **d** Architecture of VAChT. Left, viewed parallel to the membrane plane. Right, viewed from the luminal side of the vesicle. **e** Comparison of the vesamicol-bound VAChT and VMAT2 structures. Left, superimposition of the vesamicol-bound VAChT and 5-HT-bound VMAT2 (PDB ID: 8JSW) structures. Right, superimposition of the vesamicol-bound VAChT and ketanserin-bound VMAT2 (PDB ID: 8JT9) structures. The root-mean-square deviation (RMSD) between these structures are 1.2 Å and 1.1 Å, respectively. **f** Details of the interaction between VAChT and vesamicol, viewed parallel to the membrane plane. **g** Cryo-EM densities for the residues involved in vesamicol binding. **h** The binding pocket of vesamicol in VAChT and the chemical structures of benzovesamicol and trozamicol derivatives.

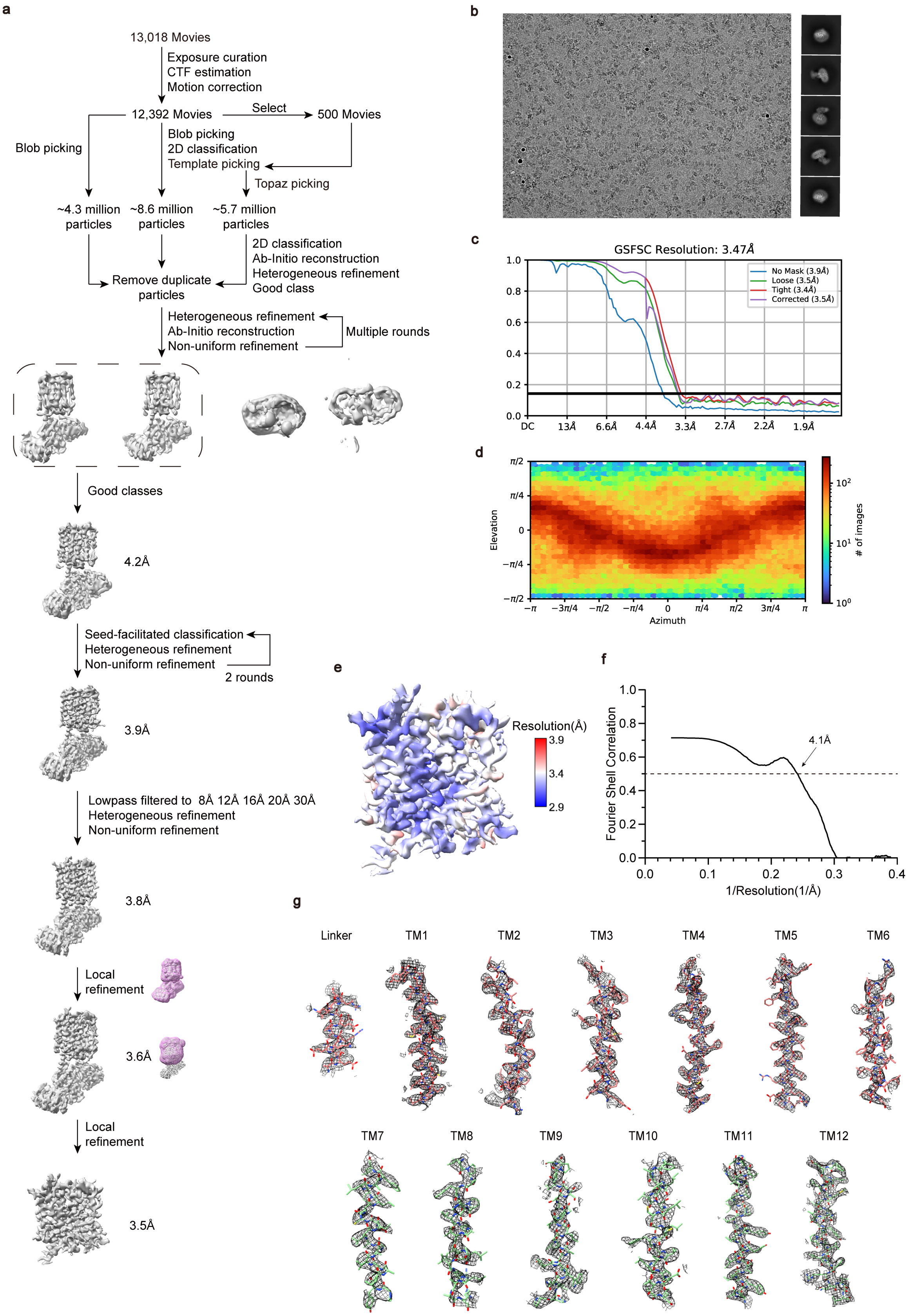
a
13,018 Movies
Exposure curation
CTF estimation
Motion correction
12,392 Movies
Select
500 Movies
Blob picking
Blob picking
2D classification
Template picking
Topaz picking
~4.3 million particles
~8.6 million particles
~5.7 million particles
2D classification
Ab-Initio reconstruction
Heterogeneous refinement
Good class
Remove duplicate particles
Heterogeneous refinement
Ab-Initio reconstruction
Non-uniform refinement
Multiple rounds
Good classes
4.2Å
Seed-facilitated classification
Heterogeneous refinement
Non-uniform refinement
2 rounds
3.9Å
Lowpass filtered to 8Å 12Å 16Å 20Å 30Å
Heterogeneous refinement
Non-uniform refinement
3.8Å
Local refinement
3.6Å
Local refinement
3.5Å
b
c
GSFSC Resolution: 3.47Å
No Mask (3.9Å)
Loose (3.5Å)
Tight (3.4Å)
Corrected (3.5Å)
DC 13Å 6.6Å 4.4Å 3.3Å 2.7Å 2.2Å 1.9Å
d
Elevation
Azimuth
# of images
e
Resolution(Å)
3.9
3.4
2.9
f
Fourier Shell Correlation
1/Resolution(1/Å)
4.1Å
g
Linker
TM1
TM2
TM3
TM4
TM5
TM6
TM7
TM8
TM9
TM10
TM11
TM12

Fig. S2 Cryo-EM data processing of the vesamicol-bound VAChT dataset.

a Summary of image processing procedures of $VAChT^{EM}$ in complex with vesamicol. All procedures were done with cryoSPARC. **b** Left, representative micrograph; right, 2D class averages. **c** Fourier shell correlation (FSC) curves between two half maps. **d** Angular distribution of particles for the final 3D reconstructions. **e** Local resolution of the cryo-EM map. **f** FSC curve calculated between the cryo-EM map and structural model. **g** Cryo-EM densities of the linker and transmembrane helices.

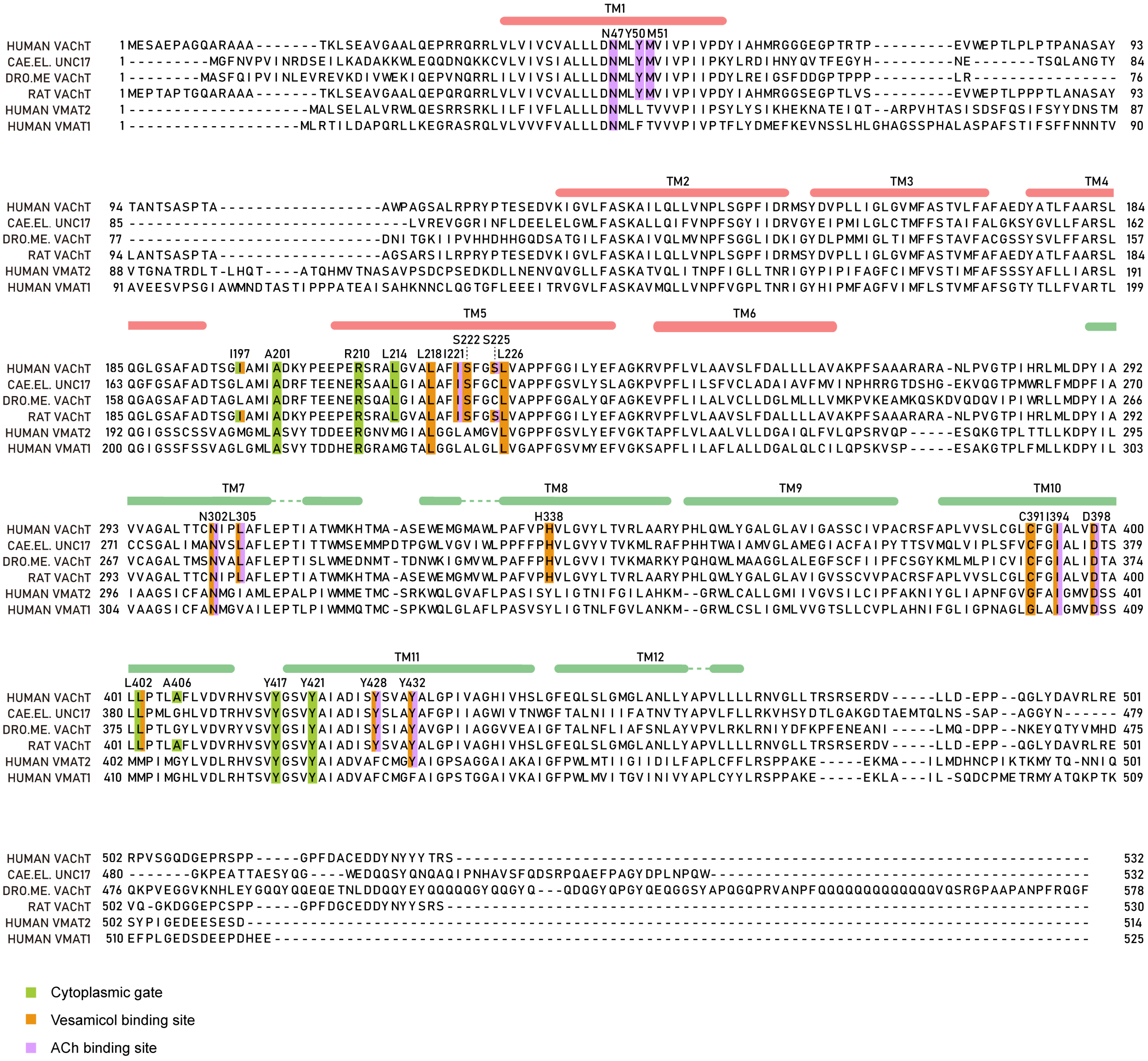

Fig. S3 Sequence alignment of the SLC18 family members.

Sequence alignment of human VAChT, caenorhabditis elegans (CAE. EL.) UNC17/VAChT, drosophila (DRO. ME.) VAChT, rat VAChT, and human VMATs (SLC18A1-2). Residues with functional roles are highlighted with different colors: green, residues forming the cytoplasmic gates; orange, residues that contribute to vesamicol binding; purple, residues that contribute to acetylcholine (ACh) binding.

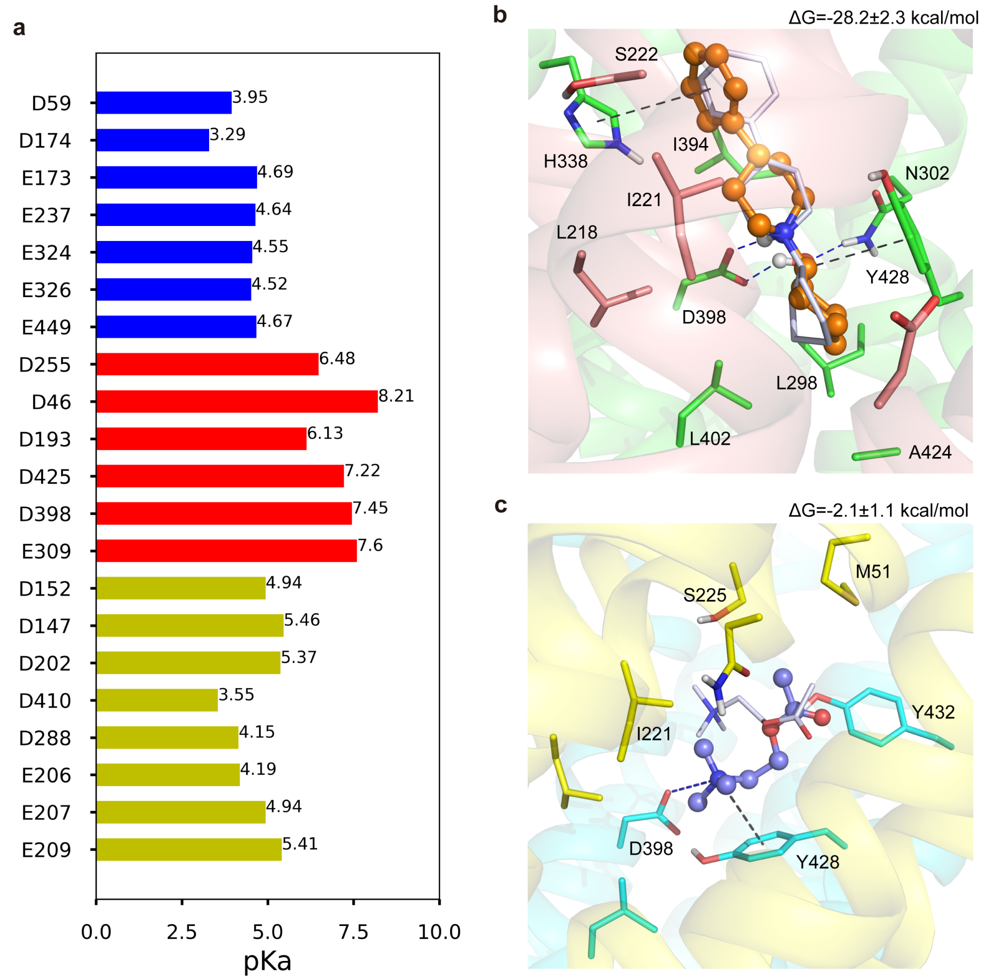

Fig. S4 Evaluation of the complex stability with MD simulations.

a Estimated pK_a values for all acidic residues in the VAChT. Here, the blue and yellow bars indicate the pK_a values of the residues on the lumen side and cytosol side, while the red bars represent the residues buried inside the membrane. **b** The binding mode between VAChT and vesamicol, with the binding free energy displayed in the top right corner. The cryo-EM structure was superimposed into the MD result and only vesamicol is shown here as gray sticks for comparison. **c** The dominant binding mode between VAChT and ACh, with the binding free energy displayed in the top right corner. For comparison, the ACh in the cryo-EM model is shown as gray sticks. Asp398 is critical for the binding to both the inhibitor vesamicol and the substrate

ACh. In the VAChT/vesamicol system (b), Asp398 not only forms a strong salt bridge with the piperidinyl group of vesamicol, but also interacts with the cyclohexanol group via a hydrogen bond. Moreover, Asn302 forms a hydrogen bond with the cyclohexanol group as well. In addition to the polar interactions, hydrophobic interactions also contribute to stabilizing the VAChT/vesamicol system. A few hydrophobic residues, including Tyr428, Ile221, Ile394, Leu218, Leu298, and Ala424, play critical roles in the binding between VAChT and vesamicol. In the VAChT/ACh system (c), on one hand, Asp398 can stabilize the binding of ACh via the charged interaction with the positive-charged choline. On the other hand, Asp398 stabilizes the position of Tyr428 by a hydrogen-bond interaction, leading to the strong cation-π interaction between the choline group and the side chain of Tyr428, which further stabilizes the binding of the ACh. In contrast to the choline group, the acetyl group in the ACh is very flexible, and no strong and long-lasting interaction was observed during the simulations, resulting in a very weak binding (-2.1 kcal/mol) between ACh and protein. Compared to the position of ACh in the cryo-EM density map, the pose identified in MD simulations showed a stronger interaction between Asp398/Tyr428 and the choline group, resulting in a shorter distance between them. We claim that the position of the ACh in the density map is acceptable, and the differences between the density map and the simulations can be attributed to the accuracy of the forcefield and the low affinity of the ACh.

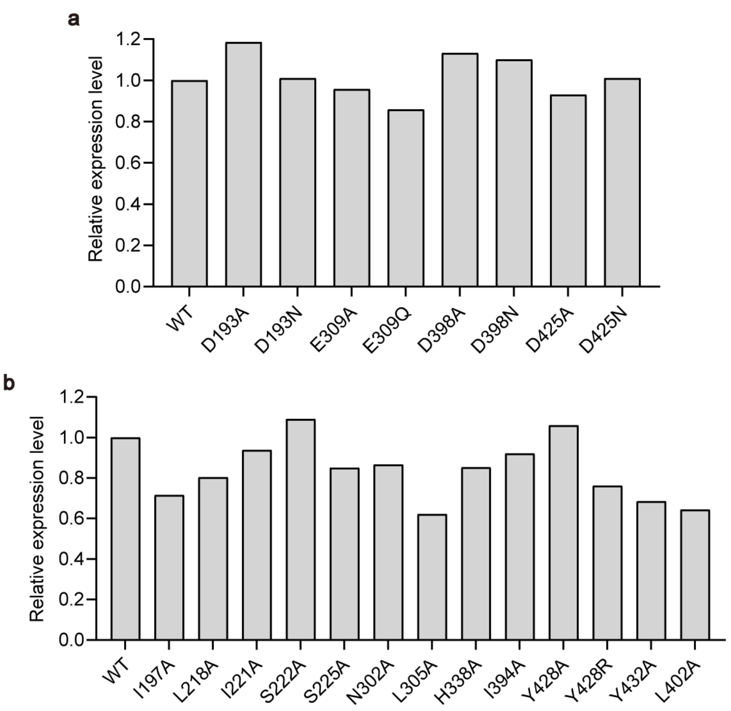

Fig. S5 Detection of the VAChT expression by FSEC.

a-b Expression levels of each VAChT mutations relative to WT VAChT.

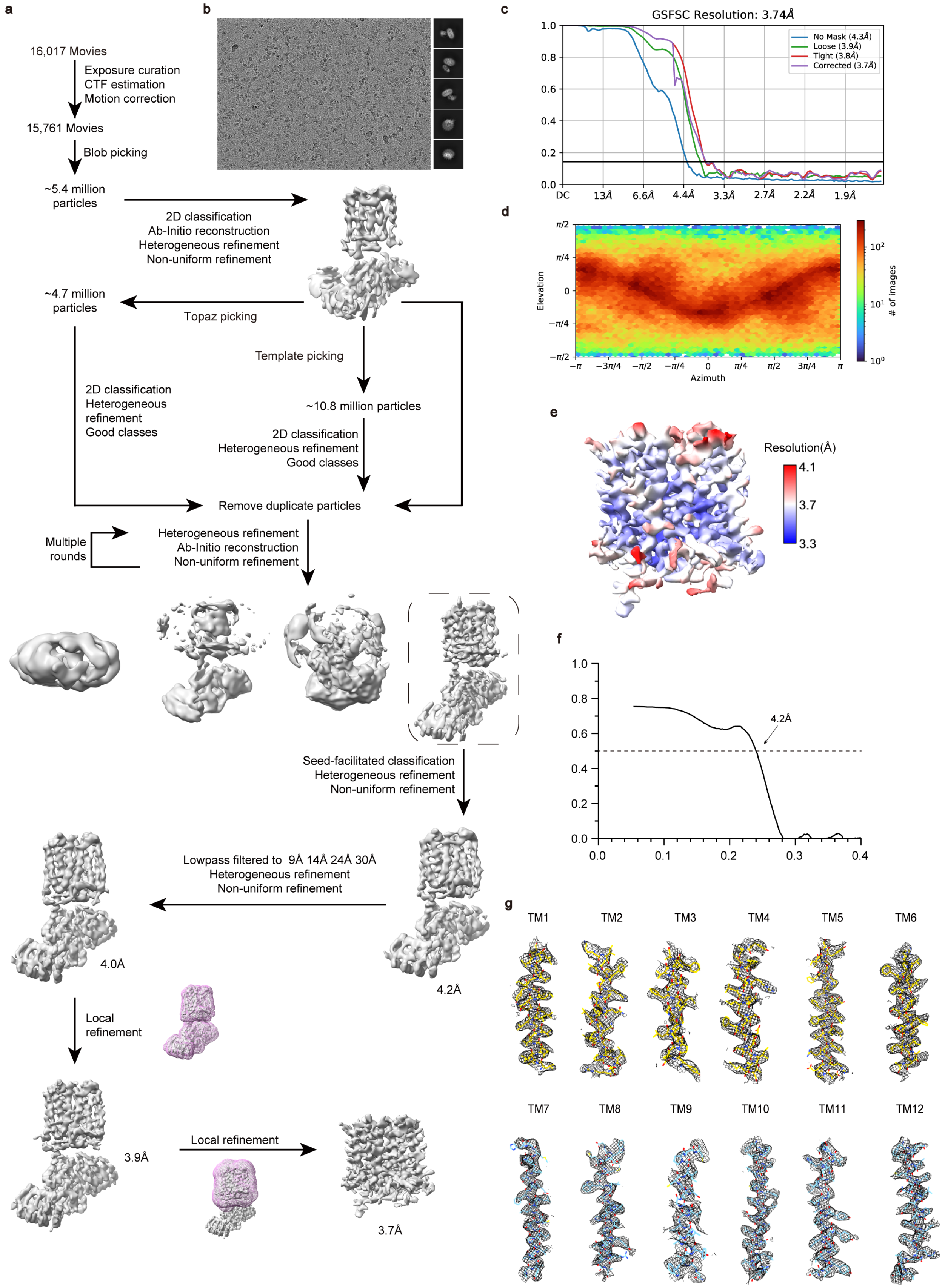
a
16,017 Movies
Exposure curation
CTF estimation
Motion correction
15,761 Movies
Blob picking
~5.4 million particles
2D classification
Ab-Initio reconstruction
Heterogeneous refinement
Non-uniform refinement
~4.7 million particles
Topaz picking
Template picking
2D classification
Heterogeneous refinement
Good classes
~10.8 million particles
2D classification
Heterogeneous refinement
Good classes
Remove duplicate particles
Multiple rounds
Heterogeneous refinement
Ab-Initio reconstruction
Non-uniform refinement
Seed-facilitated classification
Heterogeneous refinement
Non-uniform refinement
Lowpass filtered to 9Å 14Å 24Å 30Å
Heterogeneous refinement
Non-uniform refinement
4.0Å
4.2Å
Local refinement
3.9Å
Local refinement
3.7Å
b
c
GSFSC Resolution: 3.74Å
No Mask (4.3Å)
Loose (3.9Å)
Tight (3.8Å)
Corrected (3.7Å)
d
Elevation
Azimuth
# of images
e
Resolution(Å)
4.1
3.7
3.3
f
4.2Å
g
TM1 TM2 TM3 TM4 TM5 TM6
TM7 TM8 TM9 TM10 TM11 TM12

Fig. S6 Cryo-EM data processing of the ACh-bound VAChT dataset.

a Summary of image processing procedures of $VAChT^{EM}$ in complex with ACh. All procedures were done with cryoSPARC. **b** Left, representative micrograph; right, 2D class averages. **c** Fourier shell correlation (FSC) curves between two half maps. **d** Angular distribution of particles for the final 3D reconstructions. **e** Local resolution of the cryo-EM map. **f** FSC curve calculated between the cryo-EM map and structural model. **g** Cryo-EM densities of the transmembrane helices.

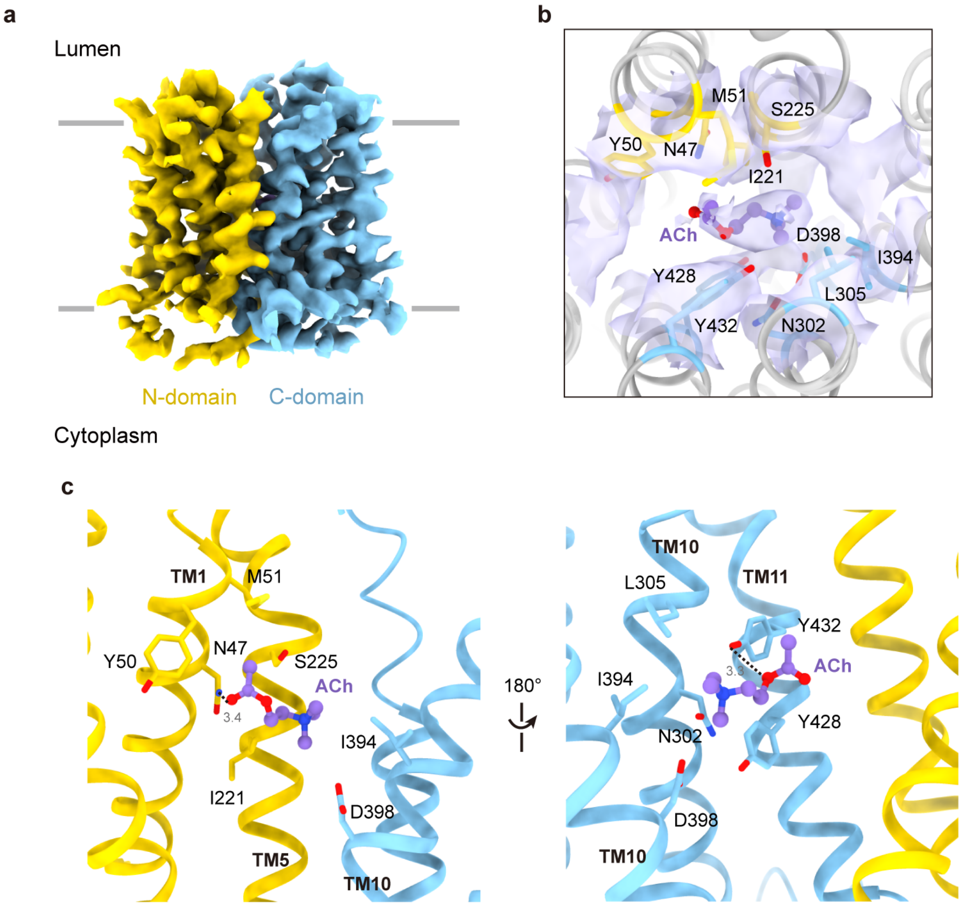

Fig. S7 Molecular mechanism of ACh recognition.

a Cryo-EM map of the VAChT/ACh complex. **b** Cryo-EM densities for the residues involved in ACh binding. **c** Details of the interaction between VAChT and ACh, viewed parallel to the membrane plane.

Table S1 Cryo-EM data collection, refinement, and validation statistics.

	Vesamicol-bound VAChT (EMD-60254) (PDB 8ZMR)	Acetylcholine-bound VAChT (EMD-60255) (PDB 8ZMS)
**Data collection and processing**		
Magnification	105,000	105,000
Voltage (kV)	300	300
Electron exposure (e^{-}/Å^2)	60	60
Defocus range (μm)	1.0-2.0	1.0-2.0
Pixel size (Å)	0.83	0.83
Symmetry imposed	C1	C1
Initial particle images (no.)	18,439,064	18,233,331
Final particle images (no.)	184,473	201,313
Map resolution (Å)	3.5	3.7
FSC threshold	0.143	0.143
Map resolution range (Å)	3.0-5.0	3.4-6.0
**Refinement**		
Model resolution (Å)	4.1	4.2
FSC threshold	0.5	0.5
Map sharpening *B* factor (Å^2)	-130.9	-199.6
Model composition		
Non-hydrogen atoms	2,916	2,920
Protein residues	384	386
Ligands	1 (Vesamicol)	1 (Acetylcholine)
B factors (Å^2)		
Protein	61.64	91.34
Ligand	54.99	74.74
R.m.s. deviations		
Bond lengths (Å)	0.009	0.007
Bond angles (°)	1.351	1.425
Validation		
MolProbity score	1.89	1.49
Clashscore	9.09	3.51
Poor rotamers (%)	0.00	0.00
Ramachandran plot		
Favored (%)	93.92	95.00
Allowed (%)	6.08	5.00
Disallowed (%)	0.00	0.00
